# Features of optical coherence tomography angiography in age-related macular degeneration with residual fluid after three loading doses of aflibercept

**DOI:** 10.1186/s40662-023-00372-z

**Published:** 2024-02-01

**Authors:** Yong-Yeon Song, Hyun-Je Seong, Jung-Tae Kim, Sung-Chul Lee, Min-Woo Lee

**Affiliations:** Department of Ophthalmology, Konyang University Hospital, Konyang University College of Medicine, #1643 Gwanjeo-dong, Seo-gu, Daejeon, Republic of Korea

**Keywords:** Age-related macular degeneration, Aflibercept, Optical coherence tomography angiography

## Abstract

**Background:**

To identify the macular neovascularization (MNV) features in exudative age-related macular degeneration (AMD) patients who exhibited residual fluid after receiving three loading doses of aflibercept.

**Methods:**

Patients were classified into two groups: Group 1, which did not exhibit intraretinal fluid (IRF) and subretinal fluid (SRF), and Group 2, which did exhibit IRF and/or SRF. Optical coherence tomography angiography (OCTA) features were assessed and compared between the groups.

**Results:**

A total of 101 eyes were enrolled; 65 for Group 1 and 36 for Group 2. No significant differences were found in baseline MNV size (2.94 ± 2.51 µm^2^ vs. 2.22 ± 2.26 µm^2^, *P* = 0.178) or vessel density (47.1 ± 15.4 % vs. 41.3 ± 10.5%, *P* = 0.052) between Groups 1 and 2. There were significant differences in the presence of loops (52.3% vs. 75%, *P* = 0.026) and peripheral arcades (29.2% vs. 55.6%, *P* < 0.001) at baseline between the two groups. In Group 1, there was a significant reduction in the presence of branching (*P* < 0.001) and loops (*P* = 0.016) after treatment. In Group 2, only the presence of branching decreased significantly (*P* < 0.001) after treatment. Multivariable analysis revealed that the presence of a peripheral arcade (B = 4.77, *P* = 0.001) was significantly associated with residual fluid.

**Conclusions:**

Although responding to treatment, the presence of loops and peripheral arcades in exudative AMD patients may contribute to residual fluid following the three loading doses of aflibercept. The peripheral arcade, in particular, may play a more significant role in the presence of residual fluid.

## Background

After the advent of anti-vascular endothelial growth factor (VEGF), the visual outcomes for patients with exudative age-related macular degeneration (AMD) have substantially improved [1, 2]. However, responses to anti-VEGF can be highly variable among patients. Generally, treatment-naïve patients with exudative AMD are recommended to receive three initial monthly injections of anti-VEGF, after which treatment strategies such as pro re nata (PRN) or treat-and-extend are typically determined. However, residual fluid after the loading phase, including subretinal fluid (SRF) and intraretinal fluid (IRF), often prompts ophthalmologists to opt for continuous treatment, influencing the course of future treatment strategies. Furthermore, such persistent fluid is associated with relatively worse visual outcomes in the long term [3].

Optical coherence tomography angiography (OCTA), allowing visualization of the retinal and choroidal vasculature noninvasively, has been reported to be useful in treating AMD patients in clinical situations. By providing visualization of macular neovascularization (MNV), OCTA facilitates the assessment of AMD patients, particularly through observation of MNV features and their responses to treatment [4, 5]. Specifically, OCTA proves to be a useful imaging modality in AMD treated with a PRN regimen of anti-VEGF by monitoring the dynamic vascular changes [6]. A previous study found that the analysis of MNV morphology using OCTA can help predict the lesion activity with good accuracy, which enables optimal decisions regarding retreatment in exudative AMD [7]. Additionally, Bae et al. [8] demonstrated that the morphological appearance of MNV on OCTA after anti-VEGF treatment can be a useful biomarker, determining whether to stop anti-VEGF injection or to continue disease suppression by maintenance therapy. However, few studies have been reported focusing on predicting the initial response to treatment in exudative AMD using OCTA. Therefore, analyzing MNV features using OCTA in patients with exudative AMD to predict the likelihood of residual fluid presence in treatment-naïve patients after the three loading doses of anti-VEGF could be insightful.

In this study, we examined the clinical features of exudative AMD patients who displayed residual fluid following three loading doses of aflibercept. We focused on both qualitative and quantitative morphological characteristics of OCT and OCTA data.

## Methods

This study was conducted in accordance with the principles of the Declaration of Helsinki and received approval from the Institutional Review Board of Konyang University Hospital, Republic of Korea (2023-01-024). Given the retrospective nature of the study, the requirement for informed consent was waived.

### Patients

We conducted a thorough review of medical records for patients aged 50 years and older who were diagnosed with treatment-naïve neovascular AMD, including type 1 and type 2 MNV, at our clinic between November 2017 and April 2022. We included patients who had received three-monthly intravitreal injections of aflibercept (Eylea^®^, Regeneron Pharmaceuticals, Tarrytown, NY, USA). Based on the OCT results obtained one month after the final injection, patients were classified into two groups: Group 1 included patients with no detectable IRF or SRF following the three loading injections, whereas Group 2 consisted of patients with detectable IRF and/or SRF. To mitigate the potential influence of age on AMD and treatment response, we structured the study to be age-matched (Group 1:Group 2 = 2:1 ratio). A total of 132 patients were initially enrolled. Of these, 31 patients were excluded from the study due to the low image quality of OCTA. As a result, a total of 101 patients were enrolled: 65 in Group 1 and 36 in Group 2. All participants underwent standard ophthalmic examinations, which included assessments of best-corrected visual acuity (BCVA), intraocular pressure (IOP), spherical equivalent (SE), and axial length using the IOL Master (Carl Zeiss, Jena, Germany). The exclusion criteria included a history of intravitreal injections or intraocular surgery except for cataract, type 3 MNV, polypoidal choroidal vasculopathy, pachychoroid neovasculopathy (PNV), any history of retinal diseases other than AMD, glaucoma, ocular trauma, as well as the presence of any other ocular diseases, including corneal diseases, retinal abnormalities, or neuro-ophthalmic diseases. If both eyes of a patient fulfilled the inclusion criteria, the eye with the better image quality was selected for more accurate analysis.

### OCT analysis

Spectral-domain OCT (SD-OCT) and OCTA were conducted using the same instrument (Spectralis OCT2; Heidelberg Engineering, Heidelberg, Germany). Two graders (YYS and MWL) evaluated the images from both groups. The graders, who were blinded to the group allocation, examined all manually measured diameters and the presence of morphological features. In case of discrepancies in the readings, resolutions were reached through open discussion. Data pertaining to central macular thickness (CMT), choroidal thickness (CT), fluid type, hyperreflective foci (HF), subretinal hyperreflective material (SHRM), pigment epithelial detachment (PED) height, and prechoroidal cleft were collected. The CMT values were measured automatically by inbuilt software. The CT was determined by averaging the measurements of the two graders, which was measured as the perpendicular distance from Bruch’s membrane to the inner surface of the sclera at the subfoveal area using software calipers in the images with enhanced depth imaging mode. Fluid type was classified into SRF and IRF. SRF was defined as the presence of fluids in the subretinal space, and IRF was defined as the presence of fluids in the retinal layers. HF was identified as discrete lesions of approximately 20–40 μm in size with increased reflectivity in the neurosensory retina. SHRM was defined as hyperreflective material situated external to the retina but internal to the retinal pigment epithelium in OCT images. The size of PED was determined by averaging the measurements of the two graders, which was measured as the vertical diameter of the largest PED in the foveal area using software calipers. CMT and BCVA at baseline, 1, 2, and 3 months after initiating treatment were recorded.

### OCTA analysis

En-face OCTA images were acquired with a 20° × 15° angle and a lateral resolution of 5.7 μm/pixel, resulting in a retinal section measuring 2.9 mm × 2.9 mm. OCTA was employed to characterize the morphological attributes of MNV. OCTA images at baseline and one month after final treatment were analyzed. Four criteria were adopted from previous studies to qualitatively describe a neovascular lesion in the images: the presence of tiny branching vessels (thin, tangled capillaries) as opposed to large mature vessels (voluminous linear filamentous capillaries); the presence of loops (internal anastomoses between tiny vessels); the presence of a peripheral arcade (connections between tiny branching vessels at the periphery); and the presence of a perilesional hypointense halo (hypointense area considered as regions of choriocapillaris alteration, corresponding to local flow impairment) [9, 10]. To quantify MNV size, the two graders independently outlined the MNV lesion using internal software. The mean values of the two measurements were used in the analysis. The vessel density (VD) of MNV was calculated using ImageJ (version 1.52a, National Institutes of Health, Bethesda, MD, USA). A free-hand selection tool was used to outline the MNV lesion in the scan, and images outside the MNV lesion were removed. The threshold adjustment tool was employed with default settings to segment grayscale images into features of interest and background. Then, the VD of the MNV was computed by dividing the area covered by white pixels by the total number of pixels. Greatest vascular caliber (GVC), representing the caliber of the main trunk or the largest vessel within the lesion area, and greatest linear dimension (GLD), representing the greatest distance enclosed by the MNV lesion area, were measured following previous studies [11, 12]. GVC and GLD were measured using the software calipers and determined by averaging the measurements of the two graders. Images with segmentation errors, motion artifacts, loss of fixation, or OCTA quality below 25 dB were excluded. In addition, images that were too ambiguous to clearly demarcate the outline of MNV at baseline or the final visit were also excluded.

### Statistical analyses

Statistical analyses were carried out using PASW Statistics (version 20, SPSS Inc., Chicago, IL, USA). The values are presented as mean ± standard deviation. For categorical variables, the Chi-squared test was employed to compare two groups. Continuous variables were analyzed using Student’s t-test. To compare changes in BCVA and CMT between the groups, repeated-measures analysis of variance (ANOVA) was conducted. A generalized estimating equation was utilized to analyze changes in the qualitative features of MNV before and after aflibercept treatment within each group. Both univariable and multivariable logistic regression analyses were used to identify factors affecting residual fluid after aflibercept injections. A *P* value of less than 0.050 was considered as statistically significant.

## Results

### Demographics

In all, 65 eyes from Group 1 and 36 eyes from Group 2 were reviewed. Table 1 shows the baseline characteristics of the patients before treatment. There were no significant differences with respect to age, sex, spherical equivalent, IOP, or BCVA between the two groups.

**Table 1:** Baseline demographics and OCT characteristics in each group

Parameter	Group 1 (n = 65)	Group 2 (n = 36)	*P* value
Age (years)	69.5 ± 7.5	70.3 ± 7.6	0.626^*^
Sex (male, %)	21 (32.3)	9 (25.0)	0.441^†^
Spherical equivalent (diopter)	0.15 ± 1.48	0.53 ± 1.14	0.183^*^
Intraocular pressure (mmHg)	13.8 ± 3.3	13.9 ± 3.5	0.788^*^
Axial length (mm)	23.6 ± 0.8	24.0 ± 0.9	0.066^*^
Baseline BCVA (logMAR)	0.53 ± 0.60	0.44 ± 0.34	0.441^*^
MNV type (1:2)	42:23	18:18	0.152^†^
CT (µm)	264.4 ± 81.3	258.9 ± 79.6	0.746^*^
CMT (µm)	390.7 ± 110.0	394.0 ± 105.7	0.885^*^
PED height (µm)	194.5 ± 157.0	168.3 ± 129.4	0.397^*^
Fluid type			0.478^†^
SRF (n, %)	39 (60.0)	26 (72.2)	
IRF (n, %)	4 (6.2)	1 (2.8)	
Both (n, %)	22 (33.8)	9 (25.0)	
Hyperreflective foci (n, %)	55 (84.6)	34 (94.4)	0.432^†^
SHRM (n, %)	23 (35.4)	17 (47.2)	0.269^†^
Prechoroidal cleft (n, %)	1 (0.5)	2 (5.6)	0.261^†^

*OCT* = optical coherence tomography; *BCVA* = best-corrected visual acuity; *MNV* = macular neovascularization; *CT* = choroidal thickness; *CMT* = central macular thickness; *PED* = pigment epithelium detachment; *SRF* = subretinal fluid; *IRF* = intraretinal fluid; *SHRM* = subretinal hyperreflective material

Data are presented as mean ± standard deviation unless otherwise stated

^*^Independent t-test; ^†^Chi-squared test

### Changes in BCVA and CMT

**Fig. 1:**
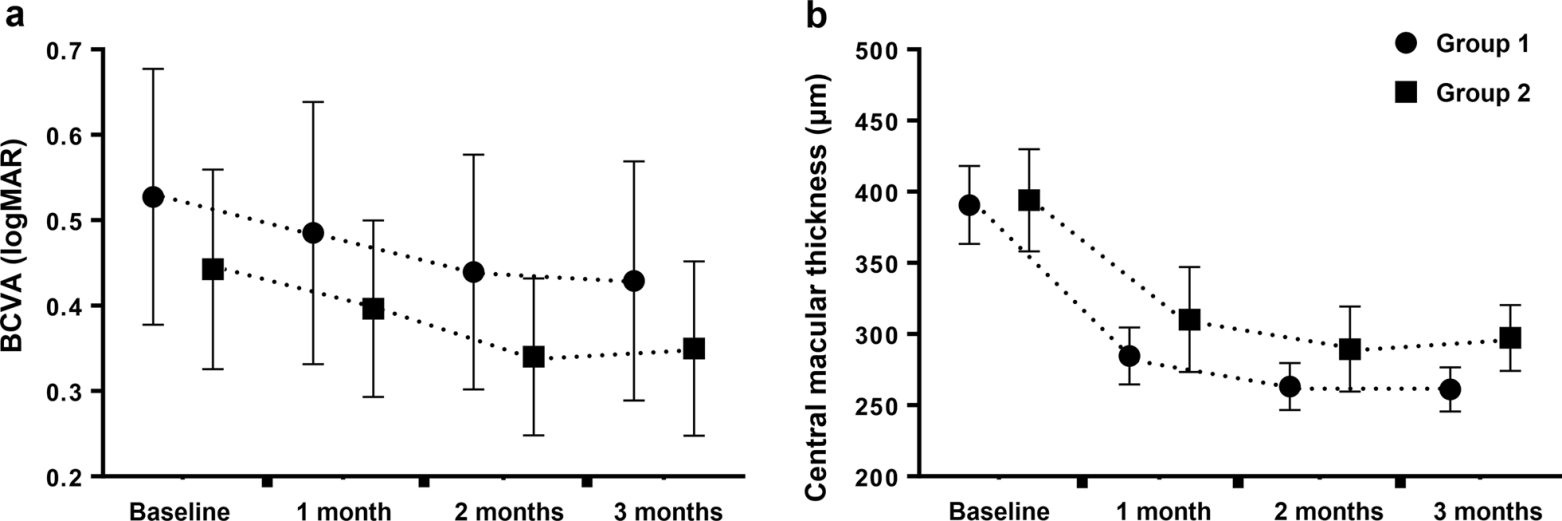
Changes in (**a**) best-corrected visual acuity (BCVA) and (**b**) central macular thickness (CMT) from baseline to one month after completion of three-loading aflibercept injections. Both groups showed significant improvements in BCVA and CMT

Figure 1 illustrates the changes in BCVA and CMT from baseline to the post-loading phase. Mean BCVA values, which were measured at baseline, 1, 2, and 3 months after initiating treatment, were 0.53 ± 0.60, 0.49 ± 0.62, 0.44 ± 0.55, and 0.43 ± 0.57 logMAR in Group 1, and 0.44 ± 0.34, 0.40 ± 0.30, 0.34 ± 0.27, and 0.34 ± 0.29 logMAR in Group 2. Both groups experienced a significant improvement in BCVA after the loading phase (both *P* < 0.001). Mean CMT values, which were measured at baseline, 1, 2, and 3 months after initiating treatment, were 390.7 ± 110. 284.6 ± 81.1, 263.2 ± 66.7, and 257.8 ± 68.8 μm in Group 1, and 396.5 ± 106.2, 310.2 ± 107.5, 289.5 ± 87, and 300.1 ± 67.3 μm, in Group 2. CMT significantly decreased in both groups after the loading phase (both *P* < 0.001). However, there were no significant differences in the changes in BCVA (*P* = 0.963) and CMT (*P* = 0.218) between the two groups, which was analyzed using repeated-measures ANOVA.

### OCT measurements

CT values were 264.4 ± 81.3 μm and 258.9 ± 79.6 μm, and the PED heights were 194.5 ± 157.0 μm and 168.3 ± 129.4 μm in Group 1 and Group 2, respectively (Table 1). Regarding fluid types, Group 1 had 39 cases with only SRF, four with only IRF, and 22 with both. In Group 2, the values were 26, 1, and 9 (*P* = 0.478). After the loading phase, Group 2 had values of 29, 5, and 2. The rate of HF was 84.6% in Group 1 and 94.4% in Group 2, while that of SHRM was 35.4% and 47.2%, respectively. There were no significant differences in OCT analysis factors between the groups. The manually measured values by the two graders, including CT and PED, showed excellent interobserver reproducibility (ICC > 0.95, CV < 5%).

### OCTA measurements

**Fig. 2:**
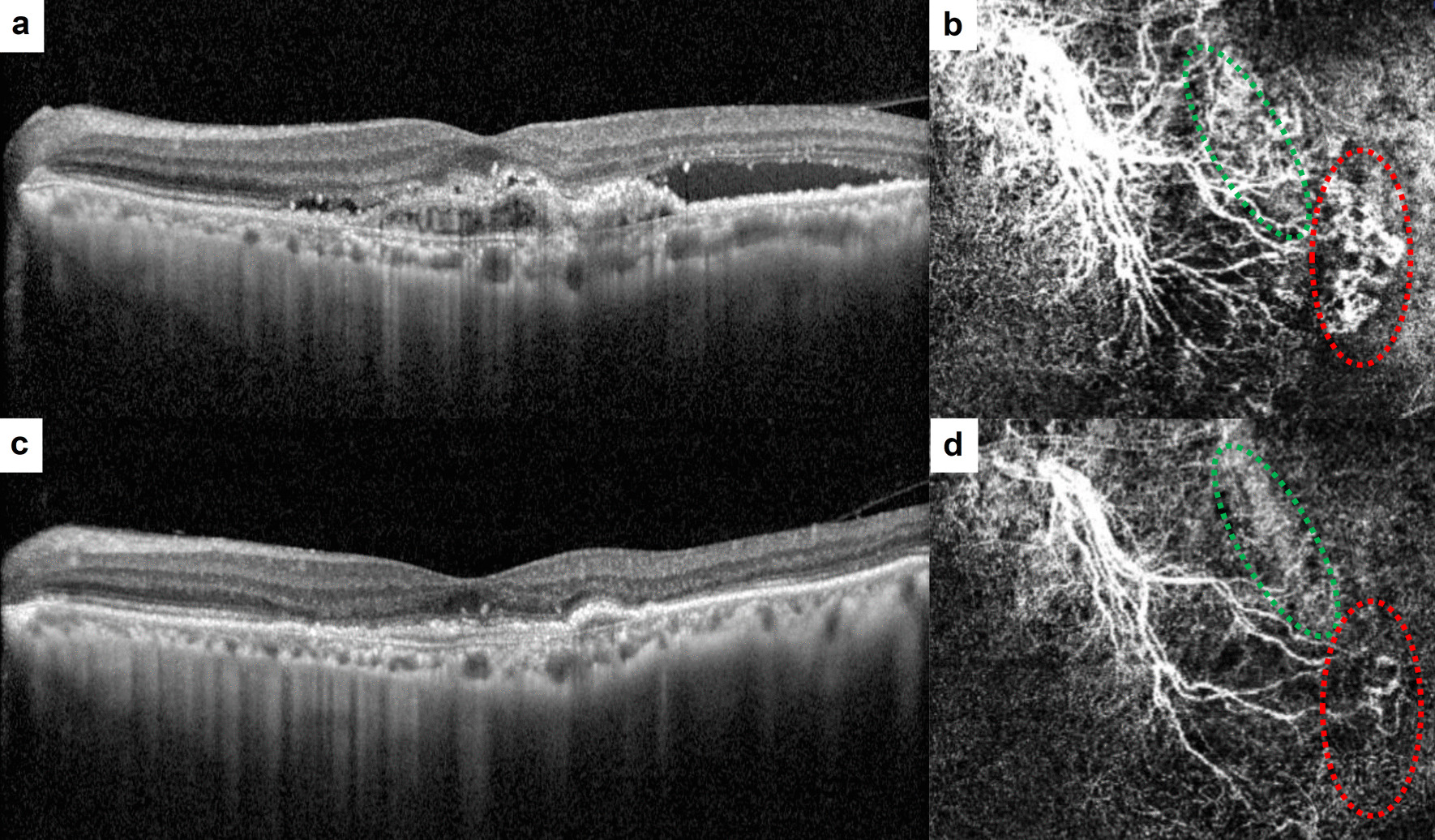
Representative images of baseline and after three loading aflibercept injections in Group 1. **a** Horizontal B-scan of optical coherence tomography (OCT) showing subretinal fluid (SRF) with pigment epithelial detachment (PED). **b** En-face OCT angiography (OCTA) shows macular neovascularization (MNV) below the PED. Note the prominent vessel with the vascular loops (green circle) and branching vessels (red circle). **c** OCT scan of the same patient after loading phase showed disappearance of SRF. **d** OCTA after loading phase showed decrease of greatest vascular caliber of the prominent vessel and overall vessel density. Note the reduction of vascular loops (green circle) and branching vessels (red circle) in the same area of the MNV.

**Fig. 3:**
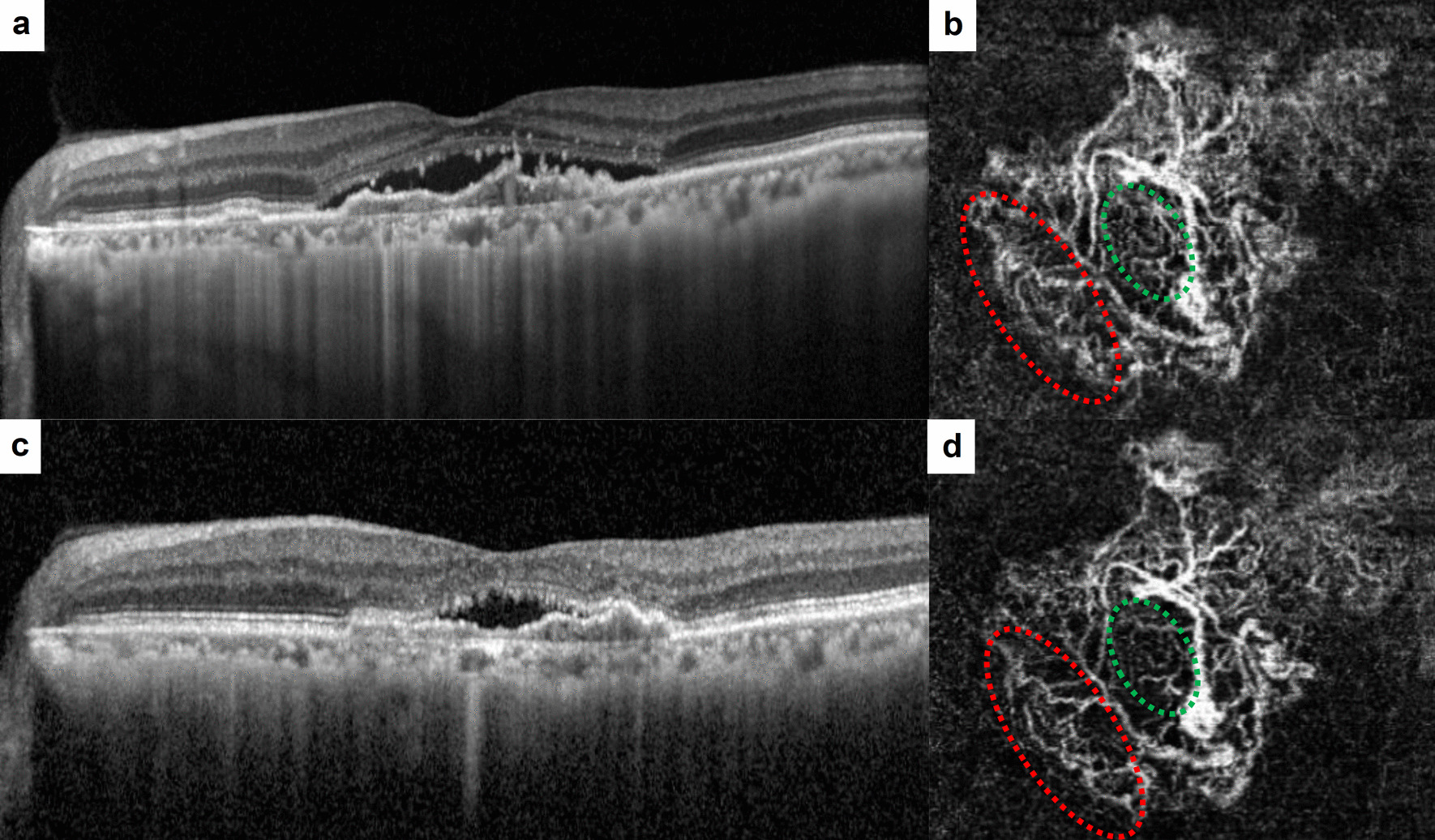
Representative images of baseline and after three loading aflibercept injections in Group 2. **a** Horizontal B-scan of optical coherence tomography (OCT) showing subretinal fluid (SRF) with pigment epithelial detachment (PED). **b** En-face OCT angiography (OCTA) shows macular neovascularization (MNV) below the PED. Note the vascular loops (green circle) and peripheral arcades of the vessel termini (red circle). **c** OCT scan of the same patient after loading phase showed remained SRF. **d** OCTA after loading phase. No noticeable changes were observed in the vascular loops (green circle) and peripheral arcades (red circle) of the same area of the MNV.

At baseline, there was no significant difference in GVC (77.0 ± 28.7 μm in Group 1 vs. 68.6 ± 30.3 μm in Group 2), GLD (2277.0 ± 963.1 μm in Group 1 vs. 1966.7 ± 952.0 μm in Group 2), MNV size (2.94 ± 2.51 mm^2^ in Group 1 vs. 2.22 ± 2.26 mm^2^ in Group 2), or VD (47.1 ± 15.4% in Group 1 vs. 41.3 ± 10.5% in Group 2) between Group 1 and Group 2 (Table 2). Within each group, all quantitative factors exhibited a significant decrease after the loading phase. Regarding the qualitative assessment of MNV, the presence of loops (52.3% in Group 1 vs. 75% in Group 2, *P* = 0.026) and peripheral arcades (29.2% in Group 1 vs. 55.6% in Group 2, *P* < 0.001) significantly differed between the groups at baseline. In Group 1, the presence of branching (*P* < 0.001) and loops (*P* = 0.016) significantly decreased after treatment (Fig. 2). In Group 2, only the presence of branching (*P* < 0.001) showed a significant reduction after treatment (Fig. 3). Similar to the OCT measurements, the OCTA values measured manually by the two graders, including GVC, GLD, and MNV size, demonstrated excellent interobserver reproducibility (ICC > 0.95, CV < 5%).

**Table 2:** Characteristics of optical coherence tomography angiography at baseline and final visit in each group

Parameter	Group 1			Group 2			
	Baseline	Final	*P* value^*^	Baseline	Final	*P* value^*^	*P* value^†^
GVC (µm)	77.0 ± 28.7	60.7 ± 23.9	**< 0.001** ^‡^	68.6 ± 30.3	59.0 ± 31.5	**< 0.001** ^‡^	0.231^§^
GLD (µm)	2277.0 ± 963.1	2011.6 ± 997.9	**< 0.001** ^‡^	1966.7 ± 952.0	1701.3 ± 969.2	**< 0.001** ^‡^	0.178^§^
MNV size (mm^2^)	2.94 ± 2.51	2.32 ± 2.19	**< 0.001** ^‡^	2.22 ± 2.26	1.65 ± 1.70	**< 0.001** ^‡^	0.178^§^
VD (%)	47.1 ± 15.4	42.4 ± 14.6	**< 0.001** ^‡^	41.3 ± 10.5	38.5 ± 10.9	**< 0.001** ^‡^	0.052^§^
Branching (n, %)	50 (76.9)	19 (29.2)	**< 0.001** ^Π^	32 (88.9)	10 (27.8)	**< 0.001** ^Π^	0.141^#^
Loop (n, %)	34 (52.3)	27 (41.5)	**0.016** ^Π^	27 (75.0)	23 (63.9)	0.093^Π^	**0.026** ^#^
Peripheral arcade (n, %)	19 (29.2)	16 (24.6)	0.253^Π^	25 (69.4)	20 (55.6)	0.087^Π^	**< 0.001** ^#^
Hypointense halo (n, %)	64 (98.5)	60 (92.3)	0.066^Π^	35 (97.2)	29 (80.6)	0.059^Π^	0.669^#^

*GVC* = greatest vascular caliber; *GLD* = greatest linear dimension; *MNV* = macular neovascularization; *VD* = vessel density

Data are presented as mean ± standard deviation unless otherwise stated

Values in boldface are statistically significant (*P* < 0.050)

^*^Comparison for baseline vs. final

^†^Comparison for Group 1 vs. Group 2 at baseline

^‡^Paired t-test; ^§^Independent t-test; ^Π^Generalized estimating equation; ^#^Chi-squared test

### Factors associated with residual fluid after the loading phase of aflibercept treatment

Univariable analysis revealed that the presence of loops (OR = 2.74; CI: 1.12 to 6.71; *P* = 0.028) and peripheral arcades (OR = 5.50; CI: 2.26 to 13.37; *P* < 0.001) were significant factors associated with residual fluid following aflibercept treatment in AMD (Table 3). In multivariable analysis, only the presence of peripheral arcades (OR = 4.77; CI: 1.82 to 12.49; *P* = 0.001) remained significantly associated with residual fluid.

**Table 3:** Logistic regression to identify factors associated with residual fluid after three loading doses of aflibercept

Parameter	Univariable		Multivariable	
	OR (95% CI)	*P* value	OR (95% CI)	*P* value
Age	1.01 (0.96, 1.07)	0.622		
Sex	0.70 (0.28, 1.75)	0.443		
Spherical equivalent	1.25 (0.90, 1.72)	0.185		
Intraocular pressure	1.02 (0.90, 1.15)	0.786		
Axial length	1.82 (0.95, 3.49)	0.073		
BCVA	0.71 (0.30, 1.70)	0.441		
MNV type	1.83 (0.80, 4.18)	0.154		
CT	0.99 (0.99, 1.00)	0.743		
CMT	1.00 (0.99, 1.00)	0.884		
HF	1.55 (0.99, 2.69)	0.118		
SHRM	1.60 (0.70, 3.66)	0.270		
PED height	0.99 (0.99, 1.00)	0.397		
GVC	0.99 (0.97, 1.01)	0.231		
GLD	1.00 (0.99, 1.00)	0.179		
Branching	2.40 (0.73, 7.88)	0.149		
Loop	2.74 (1.12, 6.71)	**0.028**	1.45 (0.52, 4.02)	0.480
Peripheral arcade	5.50 (2.26, 13.37)	**< 0.001**	4.77 (1.82, 12.49)	**0.001**
Hypointense halo	0.55 (0.03, 9.01)	0.673		
MNV size	0.87 (0.71, 1.07)	0.180		
VD	0.97 (0.94, 1.00)	0.057		

*BCVA* = best-corrected visual acuity; *MNV* = macular neovascularization; *CT* = choroidal thickness; *CMT* = central macular thickness; *HF* = hyperreflective foci; *SHRM* = subretinal hyperreflective material; *PED* = pigment epithelium detachment; *GVC* = greatest vascular caliber; *GLD* = greatest linear dimension; *MNV* = macular neovascularization; *VD* = vessel density

Values in boldface are statistically significant (*P* < 0.050)

## Discussion

The importance of OCTA is increasing for the diagnosis, treatment, and follow-up of patients with exudative AMD, and parameters related to OCTA can be used as an important biomarker to predict disease activity or prognosis in clinical situations [7, 8, 11]. Currently, the administration of an initial set of three anti-VEGF injections for AMD is strongly backed by the perspective that rapid control of neovascular activity early in the treatment course yields better visual outcomes [13–15]. In cases where residual fluid persists following the loading phase, continued injections are typically considered, as the presence of fluid is widely acknowledged as a key biomarker of exudative AMD activity [16–18]. Consequently, research that aims to predict the presence of residual fluid after a trio of aflibercept injections holds substantial value in practical clinical settings. In this study, we attempted to find factors predicting residual fluid after three loading doses of aflibercept through OCTA. This study holds practical relevance by offering insights that can help predict the need for ongoing injections. This is particularly pertinent considering that many patients harbor apprehensions about the injection procedure itself. By providing more precise information regarding the likelihood of requiring continued injections, clinicians can better manage patient expectations and alleviate concerns.

At baseline, none of the quantitative variables measured through OCTA, including GVC, GLD, MNV size, and VD, were significantly different between the two groups. Notably, these variables substantially declined after the loading phase in both groups. In McClintic et al. [19], a decrease in the size of the choroidal neovascularization (CNV) was reported in response to anti-VEGF treatment. The authors observed the reduction of vessel area and membrane area of CNV under a PRN regimen with anti-VEGF treatment. Muakkassa et al. [12]. found that MNV area and GLD using OCTA decreased after anti-VEGF treatment in treatment-naïve CNV. These findings align with our results. In our study, despite the presence of residual fluid, significant improvements in BCVA and CMT were observed after treatment in Group 2, suggesting that the MNV is responsive to aflibercept, consequently leading to reductions in the quantitative parameters that were assessed through OCTA. Therefore, these parameters in OCTA can be used as valuable markers of disease activity and response to treatment.

In terms of the qualitative features of the MNV within each group, Group 1 exhibited significant reductions in the presence of branching and loops after treatment. In contrast, Group 2 only showed a significant decrease in the presence of branching after treatment. Branching capillaries are understood to be composed of vulnerable endothelial cells without the support of pericytes, which might explain their susceptibility and reduced resistance to anti-VEGF agents [11]. Spaide et al. [11] reported that the MNV demonstrated regression of newly growing vessels following anti-VEGF injections, particularly in vessels with inadequate pericyte coverage. However, they found that vessels with pericyte coverage remained intact. This suggests that loops could represent more mature vessels, which are protected by pericytes, rather than branching capillaries. As a result, loops might exhibit a diminished response to aflibercept. Meanwhile, although the presence of loops showed a significant reduction in Group 1, no significant change in the presence of peripheral arcades occurred in either group following the loading phase. Although both loops and peripheral arcades are part of the same mechanism known as anastomosis, peripheral arcades appear to be more resistant to anti-VEGF treatment than loops. This indicates that peripheral arcades might represent a more advanced stage of pathological vessel development compared to loops. Further histopathological investigations are warranted to validate these hypotheses.

Notably, the presence of loops and peripheral arcades in the MNV was significantly higher in Group 2 than in Group 1 before treatment. Univariable analysis revealed that the presence of loops and peripheral arcades were significant factors associated with residual fluid after aflibercept treatment in AMD. However, multivariable analysis showed that only the presence of a peripheral arcade remained significant. Bae et al. [8]. noted that the closed-circuit pattern of MNV and the presence of peripheral loops were associated with the need for sustained anti-VEGF treatment. Consequently, Group 2 may contain a higher proportion of mature pathological vessels than Group 1, which could have influenced fluid absorption. Therefore, confirmation of the presence of anastomotic vessels using OCTA, especially peripheral arcade, can be useful for clinicians to predict potential partial response to anti-VEGF and to explain this to patients before treatment.

In our study, there were no significant factors associated with residual fluid following the three-injection loading phase based on baseline OCT characteristics. This contrasts with a previous study in which CT was significantly correlated with the presence of residual fluid after three loading anti-VEGF injections [10]. This discrepancy may arise from differences in inclusion criteria and anti-VEGF agents used between the two studies. Notably, the previous study employed various anti-VEGF agents and did not exclude patients with pachychoroid spectrum diseases such as PNV, which showed that CT was greater in the group with residual fluid than those without residual fluid. This study excluded eyes with PNV to reduce the heterogenicity of subjects, which resulted in CT not being significantly different between the two groups and not associated with residual fluid. The impact of CT on the response to anti-VEGF treatment may vary depending on the MNV type. Further studies including a larger number of cases with various MNV types are needed to confirm this hypothesis.

Our study has several limitations, including its retrospective design and small sample size. The investigation was based only on short-term follow-up data. Analyzing the long-term prognosis of patients with residual fluid following three aflibercept injections would be valuable. Third, the qualitative features of OCTA data are not objectively defined variables, which could result in a lot of real-world variability in analyzing each image. Fourth, our data cannot be generalized as it includes data from the Heidelberg OCTA device only, which can be different from other OCTA devices. Fifth, the confidence interval of the odds ratio, the result of multivariable analysis, is too wide. In addition, similar to other OCTA studies, our investigation was constrained to two-dimensional assessments, relying on en-face images. This limitation was due to the current technology not being able to support automated three-dimensional analyses of MNV.

## Conclusions

OCTA can provide useful information regarding residual fluid after the loading phase of aflibercept in treatment-naïve patients with exudative AMD. While there was a response to treatment, the presence of loops and peripheral arcades appears to be associated with the likelihood of residual fluid remaining after the loading phase with aflibercept. In particular, the presence of a peripheral arcade seems to be a more potent factor regarding residual fluid. Although these findings may help clinicians predict treatment responses in treatment-naïve patients with exudative AMD, the implication and clinical relevance will be determined more clearly in the future, especially with the use of swept-source OCTA.

## Data Availability

The datasets used and/or analyzed during the current study are available from the corresponding author upon reasonable request.
